# Chloroform and Ether in Puerperal Convulsions

**Published:** 1859-07

**Authors:** S. W. Burney

**Affiliations:** Forsyth Georgia


### ARTICLE III.

*Chloroform and Ether in Puerperal Convulsions.* By S. W. BURNEY M. D. Forsyth Georgia.

A contrariety of opinions are entertained in relation to the use of Chloroform in this disease. A portion of the profession speak of it in terms of eulogy and enthusiasm, whilst others are equally explicit in condemning it in every stage of uterine convulsion. To say that Chloroform will cure every case of this affection, is to speak against the record, whilst on the other hand when it is asserted with equal pertinacity and dogmatism, that it possesses no virtues whatever in controlling this fearful and dangerous disease, is running into the opposite extreme. The writer of this article has been as distrustful of this powerful agent as any of his professional brethren, whomsoever named and whithersoever located, and hence we are prepared as we think to avoid all extremes in speaking of it as a remedial agent.—We have never ventured to use it in cases of Travail, merely as an agent to lessen the suffering thereunto connected, and those who in the plenitude, of their sympathy and wisdom have thus used it, should be placed under the ban of professional censure.

But having no other desire at present, than merely to report a recent case of Eclampsia in which Chloroform and Ether acted, with as much promptitude as certainty, I shall proceed without farther prelude to the discharge of that task.

On the 30th of April last, 8 o'clock P. M., I was desired to visit Mrs. C—— who was said to be suffering greatly from an attack of sick headache. Reaching her bedside, the following facts were elicited by my interrogatories, Mrs. C—— was 8 months gone with child, temperament a due admixture of nervous and bilious, was attacked so[illegible] after dining, with vomiting and pain in the right side of head, referred principally to the 3d branch of the 5th pair of nerves.—The matter thrown up after eating her dinner was mucous and floculent and very sour to the taste, pulse but little disturbed, extremities cool, thirst urgent, bowels confined, no evidence of hyperemia about the brain. With the view of meeting these symptoms, I gave a teaspoonful of camphorated Ether and applied mustard over the stomach and extremities, and ordered a tablespoonful of Sol. Bicarb, Potass. ad. lib., the Ether and camphor was vomited instanter, which was repeated, when she began to complain of the mustard—the second dose remained with her about 5 minutes. A dose of Morphine was now given, which proved to be as ineffectual as the Camphorated Ether in controlling the sick stomach. Knowing the virtues of Dr. Fenner's precription for sick stomach I determined to give it a trial. Take of Comp., Spt., Lavender, Tinct. Opi., Spt., Camphor, equal parts Mix and give a teaspoonful every hour or two as the urgency of the symptoms may require. I now left my patient instructing Mrs. C—— to let me hear from her very early next morning, provided she remained ailing. A messenger reached my house next morning at 7, o'clock requesting me to repair to Mrs. C——'s bed side as soon as possible, but being in the country at the time, did not see her till three hours subsequently, 10 o'clock, A. M. I was informed by the family that the last prescription relieved the sick stomach, and quieted the system, enabling Mrs. C.—— to sleep from 1 o'clock till between six and seven, when she awoke in a severe *fit*. She had just gotten out of the 3d paroxysm, as I stepped into her room, her mind was wandering, countenance vacant, extremities cool, pulse 110 in the minute and not corded, stomach still sick, and still no evidence of Hyperemia of the Brain, mustard to the extremities and over the region of the stomach. Tinct Assafœtida and Valerian given at once.

This prescription was made with confidence because of the absence of the usual symptoms pointing out congestion of the brain—and when questioned, was informed by her friends that the convulsions drew her head backwards and arched her body, thus constituting opisthotonos. Soon after swallowing the physic, however, she became very restless and sick at stomach, and whilst making an effort to vomit, was attacked with her fourth convulsion. Standing by her 'till the paroxysm passed off, I was satisfied of the correctness of my diagnosis. The tinctures of Asafœtida and Valerian were to be given every hour, in suitable doses so long as the convulsions should continue; cold water to be poured on the back of the head after each paroxysm, and if not better in two hours, give sixty drops of Laudanum per anum. Urgent professional business compelled me to leave Mrs. C—— at a few moments before 11 o'clock, A. M., and was absent 'till 3 clock, P. M. The antispasmodics and Laudanum had failed to exercise any agency at all in either overcoming or lessening the convulsive throes. The patient had had two paroxysms an hour since my visit in the morning; thus proving they were gaining ground. As her pulse was now corded and resisting, and as her skin generally, was warm, with some preternatural heat about the head—I took 16 ounces of blood from the arm—which made the desired impression on the arterial system. Gave one-half grain Sulp., Morphine, and directed the nurse to keep up the cold water to the head. I was again compelled to take my leave of this case, and was absent 'till 7 o'clock, P. M. Finding no amendment in any of her symptoms, and fearing a lesion of the brain from such repeated convulsive efforts, I again repeated the use of the Lancet, especially as the pulse was as tense as a fiddle string beating 110 per minute. It required the loss of viii. ℥ of blood to make the desired impression on the pulse. It now required four strong persons to keep the patient on the bed, and as I supposed the extreme jactitation was in part owing to labor pains, I examined her per vaginam. The os was found fully dilated, and the membranes protruding. Ruptured the membranes, and had her placed in position for delivery. The womb continued to contract at proper intervals, and during those propulsive efforts, the patient would scream and make strong efforts to get off her bed. So my post and that of four assistants was no sinecure. Her labor terminated at 9 o'clock, giving birth to a still born child. Efforts were made to resuscitate it, though without success. The Placenta being somewhat too adherent, it required an hour to detach it without resorting to the introduction of the hand into the uterus; the moment the placenta was taken away, she had another convulsion, the severest in the opinion of her mother, with which she had been affected, I now took of Chloroform and Sulp., Ether—aa—equal parts, and poured about ii℥ upon a pocket handkerchief, and applied it to her nose and mouth, occasionally recruiting until the patient fell into a sweet and refreshing sleep. She slept for two hours, when she awoke very noisy and restless, the anesthetic was again resorted to with the same prompt relief. I now went to bed directing the nurse proper to repeat the remedy whenever the patient manifested any sign of restlessness. It was used twice between the hour of my retiring and day break, always quieting the system and warding off an attack. The patient had no paroxysm after this remedy was resorted to—awoke at 9 o'clock next morning more conscious than at any time since her attack. It was now found an easy matter to keep her in a proper condition with Assafœtida and Valerian—opening the bowels and drawing off the urine with the catheter. She mended apace—is now able to set up, and eat her allowance. She has no recollection of any thing that transpired from the morning of the day she was attacked with sick headache 'till her consciousness was completely regained.

